# The involvement of spontaneous brain activity in natural recovery from internet gaming disorder: A resting-state fMRI study

**DOI:** 10.3389/fpsyt.2023.1093784

**Published:** 2023-02-21

**Authors:** Xiaoyue Liu, Yawen Zheng, Michelle Niculescu, Qi Liang, Ai Yang, Guangheng Dong, Zhonghui Gao, Ping Lin, Yanlong Liu, Li Chen, Danjun Xu

**Affiliations:** ^1^School of Mental Health, Wenzhou Medical University, Wenzhou, China; ^2^Lishui Second Affiliated Hospital of Wenzhou Medical University, Lishui, China; ^3^Department of Social Sciences, Chatham University, Pittsburgh, PA, United States; ^4^Centers for Cognition and Brain Disorders, Hangzhou Normal University, Hangzhou, Zhejiang, China; ^5^The Affiliated Xiangshan Hospital of Wenzhou Medical University, Ningbo, China

**Keywords:** internet gaming disorder, natural recovery, resting-state fMRI, regional homogeneity, reward function

## Abstract

**Objective:**

Internet gaming disorder (IGD) can seriously impair an individual’s physical and mental health. However, unlike the majority of those suffering from substance addiction, individuals with IGD may recover without any professional intervention. Understanding the brain mechanisms of natural recovery from IGD may provide new insight into how to prevent addiction and implement more targeted interventions.

**Methods:**

Sixty individuals with IGD were scanned by using a resting-state fMRI to assess brain region changes associated with IGD. After 1 year, 19 individuals with IGD no longer met the IGD criteria and were considered recovered (RE-IGD), 23 individuals still met the IGD criteria (PER-IGD), and 18 individuals left the study. The brain activity in resting state between 19 RE-IGD individuals and 23 PER-IGD individuals was compared by using regional homogeneity (ReHo). Additionally, brain structure and cue-craving functional MRIs were collected to further support the results in the resting-state.

**Results:**

The resting-state fMRI results revealed that activity in brain regions responsible for reward and inhibitory control [including the orbitofrontal cortex (OFC), the precuneus and the dorsolateral prefrontal cortex (DLPFC)] was decreased in the PER-IGD individuals compared to RE-IGD individuals. In addition, significant positive correlations were found between mean ReHo values in the precuneus and self-reported craving scores for gaming, whether among the PER-IGD individuals or the RE-IGD individuals. Furthermore, we found similar results in that brain structure and cue-craving differences exist between the PER-IGD individuals and RE-IGD individuals, specifically in the brain regions associated with reward processing and inhibitory control (including the DLPFC, anterior cingulate gyrus, insula, OFC, precuneus, and superior frontal gyrus).

**Conclusion:**

These findings indicate that the brain regions responsible for reward processing and inhibitory control are different in PER-IGD individuals, which may have consequences on natural recovery. Our present study provides neuroimaging evidence that spontaneous brain activity may influence natural recovery from IGD.

## Introduction

Internet gaming is a legitimate leisure activity worldwide; However, there are emerging concerns due to the high proportion of gamer that become addicted. Thus, Internet Gaming Disorder (IGD) was added to the latest version of the Diagnostic and Statistical Manual of Mental Disorders (DSM-5) as a condition warranting further research (1). Proposed as a behavioral addiction (2), IGD shares many similarities in both physical and psycho-social manifestations with substance use disorder, including aspects of tolerance, withdrawal, repeated unsuccessful attempts to cut back or quit, and impairment in everyday life functioning (3).

Although IGD has been repeatedly linked to a wide range of detrimental psychological and health consequences, one phenomenon attracting researchers’ attention is that individuals with IGD may recover without professional intervention (4, 5) and the recovery rates range from 36.7 to 51.4% (6, 7). For example, King et al. (4) tracked 117 pathological online game users and found that the symptoms of addiction were alleviated without any intervention after 18 months (4). Later, a follow-up study with 1545 participants that were addicted to the internet had a similar finding that half went into remission and no longer met the diagnostic criteria for IGD without any obvious intervention (5).

Unlike substance addiction, IGD has a unique feature. While neurotransmission is affected by gaming, it is not due to a foreign substance like other addictions. Therefore, individuals with IGD may find it easier to recover naturally over time without any intervention than those that suffer from SUDs. Consequently, it seems that there are certain mechanisms underlying the self-remission of IGD. Previous studies on IGD have demonstrated that this self-remission may be associated with psychological and social factors (5, 7). For instance, psychological factors such as loneliness, depression, anxiety, low self-esteem, and low level of social support all affect the natural recovery from IGD. During a cue-craving task, researchers found that the brain regions (including the lentiform, DLPFC, and insula) were activated differentially in those with persistent IGD when compared to those that recovered from IGD (8). However, it is not clear whether the neural mechanism in resting-state affects the natural recovery from IGD. An improved understanding of neural factors relating to recovery in IGD may provide new insight into how to prevent addiction and implement more targeted interventions.

Resting-State fMRI (rs-fMRI) is a key method widely used to explore brain mechanisms of IGD (9). Resting state is that individuals remains relaxed, stationary, eyes closed, and avoid any systematic thinking. It can reflect the spontaneous brain activity and permanent changes on brain, which may be more representative of long-term changes and has important physiological significance (10). Regional homogeneity (ReHo) is often used to evaluate brain activity synchronization in resting-state of healthy populations and patients (11). It is a rank-based non-parametric data-driven approach that reflects the temporal homogeneity of the regional BOLD signal. Due to independence of the onset time of stimulus, this method is useful for the analysis of resting-state fMRI data (10). Although the physiological and some preprocessing impacting the measure, the test-retest reliability of ReHo has been found to be very high (12). The high test-retest reliability allows ReHo for exploring the changes of functional homogeneity in some diseases, as well as in addiction behaviors.

Resting-state fMRI revealed that those addicted to internet gaming showed significantly enhanced activity in the cingulate gyrus, middle frontal gyrus, precuneus, and posterior central gyrus, and these brain regions may be related to the reward function (13). Our previous study found individuals with IGD have stronger reward network connection and lower inhibition and control ability (14). A large number of relevant studies found that the brain regions related to reward processing and inhibitory control [including the dorsolateral prefrontal cortex (DLPFC), precuneus, orbitofrontal cortex (OFC), cingulate gyrus, etc.] are impaired in internet game addicts (15–19). All the above study results indicate that reward sensitivity in IGD individuals is enhanced, accompanied by the weakening of inhibition and control ability. Studies on substance addiction have found that non-abstinent users show a high craving for substance-related cues associated with brain functional and structural changes compared with abstinent individuals. Further, these brain regions may affect the subsequent substance withdrawal (20–22).

However, whether these changes in brain regions and functions are related to the development and maintenance of IGD requires further research. In the current study, we compared data on resting-states between those who subsequently recovered (RE-IGD) versus persistent (PER-IGD). At the same time, we analyzed the structural and cue-craving data of the individuals with RE-IGD and PER-IGD to further confirm our results. Considering previous neuroimaging data about IGD, we speculated that the brain regions related to reward processing and inhibitory control may be impaired in PER-IGD individuals compared to RE-IGD individuals.

## Materials and methods

### Ethics

This study complied with the Declaration of Helsinki and was approved by the Ethics Committee of Wenzhou Medical University. All recruited subjects were university students from Zhejiang province, China. All participants provided written informed consent before participation.

### Internet gaming disorder

Young’s Internet addiction test (IAT) (23) and the nine DSM-5 criteria of IGD (1) were used together to select IGD participants. Actually, many studies have used DSM-5 to diagnose the IGD and its accuracy has been proved (24–26). In our study, we used DSM-5 and IAT together, and the criteria are more strict. Participants were diagnosed with IGD if they satisfied the following criteria: (1) scored 50 or higher on the IAT scores; (2) met at least five DSM-5 criteria; and (3) played online games at least 5 of 7 days in a week (frequency) and more than 14 h (amount) per week in the past two years. All participants underwent structured psychiatric interviews [Mini-International Neuropsychiatric Interview (MINI)] conducted by an experienced psychiatrist, and individuals with psychiatric or neurological disorders were excluded. In addition, no subjects reported previous experience with gambling or illicit drugs (e.g., cannabis and heroin). All participants were instructed not to use any medicine or substances including coffee, tea, and alcohol on the day of scanning.

### Recovery from and persistence of IGD

We first recruited and scanned 60 IGD participants, and one year later, we surveyed these IGD participants again. Of the returning participants, 42 agreed to continue participation. Twenty-three (9 females) participants still met IGD criteria (PER-IGD) and nineteen (5 females) no longer met IGD criteria (RE-IGD).

### Co-variables

Age, education level and gender were treated as covariates. We also explored environmental factors (including family support, changes in romantic relationships, occupation, free time, study habits, health problems, aspects of gaming, and opinions regarding technology) during the year between visits by use a tracking survey to exclude its influence on our results (Supplementary material). In addition, some participants still met the IGD criteria although their IAT scores decreased significantly. Therefore, we will take the changes of IAT scores between the first and the second test and the other possible influencing factors as co-variables for the statistical analysis as control for extraneous variables.

### Experiment procedure and data collection

First, considering that craving is the core symptom of IGD and affects the withdrawal associated with addiction (27), we used the self-reported craving scale to collect subjects’ feelings about their craving. The questionnaire was adapted from the Questionnaire of Smoking Urges (28) and consists of 10 items measuring urges on a scale from 1 (low) to 10 (high). Then, functional MRI scans were performed on a 3.0 Tesla Siemens Trio scanner for the initial recruited 60 IGD subjects. All subjects were asked to rest quietly in the scanner and not fall asleep. The data was acquired using gradient echo planar imaging sequence with the following parameters: [repeat time (TR) = 2s, flip angle = 90°, echo time (TE) = 30 ms; 33 slice per volume; interleaved sequence; 3 mm thickness; matrix 64 cm × 64 cm, field of view (FOV) = 220 mm × 220 mm, acquisition matrix = 64 × 64]. The scan last for 7 min for each participant and each functional run included 210 imaging volumes. One year later, we surveyed these IGD participants again by DSM-5 and IAT questionnaires, and collected 23 PER-IGD participants and 19 RE-IGD.

### Data pre-processing

We used Data Processing Assistant for Resting-State fMRI (DPARSFA)^1^ to conduct data pre-processing (29). The first 10 volumes of each functional time series were abandoned to avoid the instability of the initial fMRI signal, thereby leaving 200 volumes to be analyzed. Then, we conducted pre-processing with the data, which included slice timing, head motion correction, and spatial normalization to a standard template. Participants were excluded if their head data exceeded 2.5 mm or maximum rotation that exceeded 2.5°. To reduce the effects of confounding factors, a regression of nuisance signals including cerebral spinal fluid, white matter, and six motion vectors and detrending was performed. The temporal filtering (0.01–0.08 Hz) was used to the time series of each voxel to reduce low-frequency drift and high-frequency noise.

### ReHo analysis

The preprocessed data was used for ReHo analysis with DPARSFA. Individual ReHo maps were generated by calculating the KCC of the time series of a given voxel with its nearest neighbors (26 voxels) in all directions on a voxel-wise basis. The calculated formula of ReHo is defined as follows:


(1) $$W=\frac{\sum {\left(R_{i}\right)}^{2}-n\,{\left(\bar{R}\right)}^{2}}{\frac{1}{12}\,K^{2}\,\left(n^{3}-n\right)}$$

Where W (ranging from 0 to 1) is the KCC among time series of given voxel. *R*_*i*_ is sum rank of the *i*th time point; n is the number of ranks; $\bar{R}$ = ((n+1) K)/2 is the mean of the *R*_*i*_; *K* is the voxel number among time series. To reduce the influence of individual variations in the KCC value, each standardized ReHo map was generated by dividing the raw ReHo map by the global mean ReHo. Spatial smoothing was conducted on the ReHo maps with a Gaussian kernel of 4 mm × 4 mm × 4 mm full-width at half-maximum (30).

### Statistical analysis

To explore the brain activity differences between the RE-IGD individuals and the PER-IGD individuals, a two-sample *t*-test was performed on the normalized ReHo with REST software. The result and statistical map were set at a combined threshold of *p* < 0.05 (AlphaSim corrected) and a minimum cluster size of 135 voxels.

To evaluate the association of altered ReHo in different brain regions, we performed Spearman’s correlation analysis between mean ReHo values and self-reported craving scores and IAT scores of PER-IGD individuals and RE- IGD individuals. Correlations between brain response features and scale scores can help us better understand the main findings.

### Structural and functional MRI collection and analysis

Scanning was also performed on a 3.0 Tesla Siemens Trio scanner for these recruited 60 IGD subjects. In this study, we used a cue-craving task to explore the brain activities about these IGD participants. Considering the functional fMRI results are only a supplement to our study, we have not given a detailed description, for details, please refer to our previous research (27).

The Functional MRI data was acquired using gradient echo planar imaging sequence with the following parameters: interval scanning 33 slices, 3 mm thickness, TR = 2,000 ms, TE = 30 ms, flip angle = 90°, field of view = 220 mm × 220 mm, matrix = 64 × 64. Structural data covering the whole brain was acquired using a T1-weighted three-dimensional spoiled gradient-recalled sequence (176 slices, TR = 1,700 ms, echo time (TE) = 3.93 ms, slice thickness = 1.0 mm, flip angle = 15°, field of view (FOV) = 240 mm × 240 mm, in-plane resolution = 256 × 256). The functional MRI data were analyzed using SPM12 (Statistical Parametric Mapping)^2^ (31). Structural data were analyzed using Freesurfer,^3^ which is software developed by Harvard University (32). Considering these data are only supplementary, the specific analysis process and cue-craving task will not be described in detail in the present study. Our previous research studies have specific descriptions available for reference (27, 33).

## Results

### Demographic features between RE-IGD and PER-IGD

In the study, we recruited and scanned a total of 60 IGD participants. One year later, we surveyed these IGD participants again and 42 participants agreed to join our study again. With the 42 participants, 23 (9 females) were classified as PER-IGD and 19 (5 females) as RE-IGD individuals. There are significant differences between RE-IGD individuals and PER-IGD individuals on track IAT score (RE-IGD: 41.37 ± 9.57, PER-IGD: 64.96 ± 9.57; *t* = 7.87, *p* < 0.000), and track DSM-5 score (RE-IGD: 1.89 ± 1.10, PER-IGD: 5.39 ± 1.73; *t* = 7.64, *p* < 0.000), and tracked game-playing per week (RE-IGD: 11.84 ± 8.28, PER-IGD: 20.57 ± 10.97; *t* = 2.86, *p* = 0.007). No differences were found in terms of demographic information and general information before IGD between these two groups (Table 1).

**TABLE 1:** Demographic information.

	RE-IGD (*n* = 19)	PER-IGD (*n* = 23)	*t*	*p*
Age, Years (mean ± SD)	21.32 ± 2.03	20.83 ± 2.21	−0.74	0.463
Education (years) (mean ± SD)	14.11 ± 2.35	13.48 ± 2.56	−0.82	0.419
IAT score (mean ± SD)	60.63 ± 8.09	64.91 ± 11.02	1.41	0.167
DSM-5 score (mean ± SD)	5.63 ± 1.30	5.61 ± 1.12	−0.06	0.951
Game playing per week (hours) (mean ± SD)	18.89 ± 10.09	17.61 ± 6.99	−0.49	0.629
Track IAT score (mean ± SD)	41.37 ± 9.57	64.96 ± 9.75	7.87	0.000***
Track DSM-5 (mean ± SD)	1.89 ± 1.10	5.39 ± 1.73	7.64	0.000***
Track gaming playing per week (hours) (mean ± SD)	11.84 ± 8.28	20.57 ± 10.97	2.86	0.007**

IAT, internet addiction test; DSM, diagnostic and statistical manual of mental disorders; SD, standard deviation. ***p < 0.001; **p < 0.01.

### Brain region results

**FIGURE 1:**
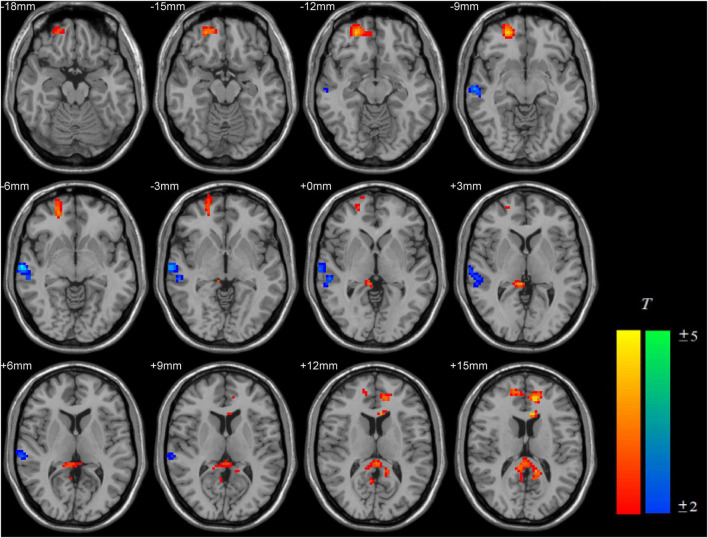
Brain areas with increased and decreased ReHo in PER-IGD individuals compared with RE-IGD individuals. Two-sample *t*-test, *p* < 0.05, AlphaSim corrected, voxel size = 3 × 3 × 3; T-score bars are shown on the right bottom. The voxels with hot colors represent increased ReHo in the PER-IGD individuals, and cold colors indicate decreased ReHo in the PER-IGD individuals.

Compared with the RE-IGD individuals, the PER-IGD individuals showed increased ReHo values in the brain regions responsible for reward including the DLPFC, the OFC and the precuneus. The ReHo values in the middle temporal gyrus and the postcentral gyrus of the PER-IGD individuals were decreased compared to RE-IGD individuals. There were no significant differences in other brain regions between these two groups. Table 2 and Figure 1 showed the detail information for the brain regions with ReHo difference between RE-IGD individuals and PER-IGD individuals.

**TABLE 2:** Brain areas showing Regional homogeneity (ReHo) difference between recovered IGD and persistent IGD.

Areas	x	y	z	voxels	BA	*t*
**PER-IGD > RE-IGD**						
R orbitofrontal cortex	24	51	−12	136	11	3.76
L precuneus	−12	−54	30	345	31	4.32
L dorsolateral prefrontal cortex	−9	42	18	192	9	4.25
**PER-IGD < RE-IGD**						
R middle temporal gyrus	66	−15	−12	136	21	−3.78
L postcentral gyrus	−36	−24	45	270	3	−3.20

Voxel size = 3mm × 3mm × 3mm, *p* < 0.05, AlphaSim corrected and at least 136 voxels. L, left; R, right; x, y, z represent peak MNI coordinates; BA, Brodmann area; *t*, *t*-values from a two-sample *t*-test of the statistical different clusters. The brain regions were referenced to the software Xjview (http://www.alivelearn.net/xjview/) and verified through comparisons with a brain atlas.

### Correlation results

**FIGURE 2:**
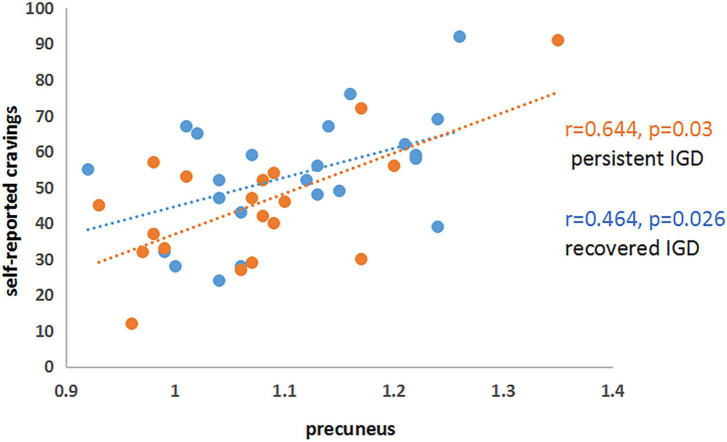
Correlations between precuneus and self-reported cravings in PER-IGD and RE-IGD groups. Correlations between precuneus and self-reported cravings in PER-IGD and RE-IGD groups.

Significant positive correlations were found between mean ReHo values in the precuneus and self-reported craving to gaming among PER-IGD individuals (*r* = 0.644; *p* = 0.03) and RE-IGD individuals (*r* = 0.464; *p* = 0.026) (Figure 2), respectively. There was no significant correlation between addiction scores and other brain regions.

### Functional MRI and structural results

**FIGURE 3:**
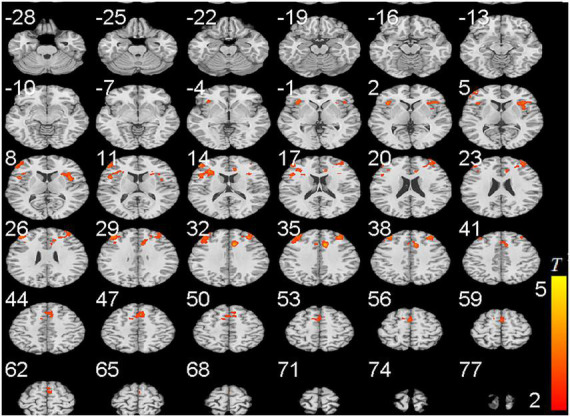
Brain areas activation showing differences in two groups (PER-IGD - RE-IGD). Two-sample *t*-test, *p* < 0.01, FWE corrected, voxel > 90; T-score bars are shown on the right bottom. The voxels with hot colors represent higher activation of brain regions in the PER-IGD individuals.

To further confirm these results, we collected cue-craving task functional MRI and structural data. We found that the activities of DLPFC, anterior cingulate gyrus, and insula were significantly higher in the PER-IGD individuals compared to RE-IGD individuals (Table 3 and Figure 3).

**TABLE 3:** Brain areas showing difference between individuals with RE-IGD and PER-IGD.

Areas	x	y	z	voxels	BA	*T*
**PER-IGD > RE-IGD**						
L dorsolateral prefrontal cortex	−42	45	30	304	46	4.03
R anterior cingulate gyrus	9	18	33	211	32	4.43
R dorsolateral prefrontal cortex	33	39	36	134	9	3.96
R insula	39	9	6	97	13	3.51

L, left; R, right; x, y, z represent peak MNI coordinates; BA: brodmann area; FWE corrected, *p* < 0.01, voxels > 90.

**FIGURE 4:**
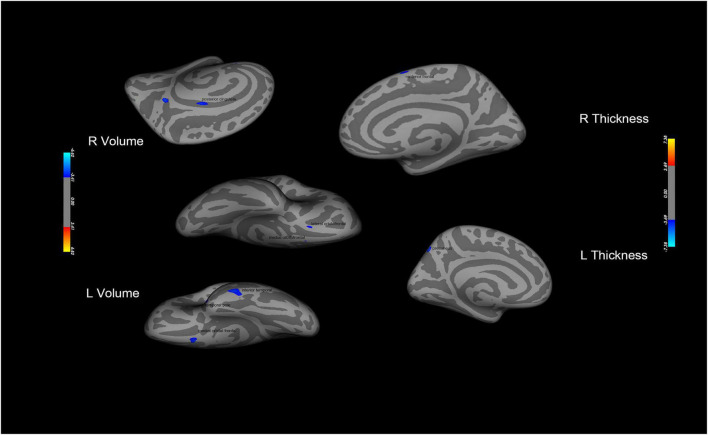
Brain areas with significant differences in gray matter volume or thickness between the two groups (PER-IGD−RE-IGD). Two-sample *t*-test, *p* < 0.05, FDR corrected; T-score bars are shown on the right bottom. The voxels with blue colors represent low gray matter volume or thickness in the PER-IGD individuals.

The results of structural data showed that the gray matter volume or thickness in several regions including the OFC, precuneus, and superior frontal gyrus were decreased in the PER-IGD individuals compared to RE-IGD individuals (Table 4 and Figure 4).

**TABLE 4:** Brain areas showing differences in gray matter volume or thickness between the two groups (PER-IGD − RE-IGD).

Areas	x	y	z	Size (mm^2^)	*T*
R posterior cingulate gyrus	7.1	−15.8	30.3	33.30	−4.09
L medial orbitofrontal cortex	−6.6	16.8	−13.8	37.54	−3.77
R lateral orbitofrontal cortex	15.7	20.7	−23.7	12.01	−3.61
R medial orbitofrontal cortex	6.1	19.9	−18.2	3.09	−3.43
R precuneus	5.7	−56.4	18.8	52.82	−4.53
L Inferior temporal	−53.7	−19.9	−31.1	117.62	−3.83
L temporal pole	−41.2	14.8	−31.8	29.45	−3.71
L precuneus (thickness)	−10.9	−75.1	42.7	62.44	−5.08
R superior frontal (thickness)	9.4	6.8	65.7	64	−3.45

L, left; R, right; x, y, z represent peak MNI coordinates; size: largest surface area of activation voxel block; *p* < 0.05, FDR corrected.

## Discussion

The current study explored the potential factors that affected natural recovery from IGD by spontaneous brain activity in resting-state. We investigated 60 IGD individuals and followed up 19 individuals with IGD who subsequently recovered and 23 individuals who did not recovery one year later. Our findings largely supported our priori hypotheses that brain regions related to reward processing and inhibitory control may affect recovery from IGD. Implications of this study findings are discussed below.

### Increased ReHo values in reward -related regions in the PER-IGD individuals

This study found increased ReHo values in the orbitofrontal cortex and the precuneus of the PER-IGD individuals compared with RE-IGD individuals. The ReHo values mainly reflect the consistency of the activities of brain regions, representing the enhancement or weakening of the connections (10). First, the OFC is essential for selecting goals based on current, updated values of expected reward outcomes (34). It generates and maintains its reward expectation and plays an important role in reward processing (35). Second, the precuneus plays a critical role in visual processing, attention, and tracking stimuli (36, 37). Furthermore, previous research indicates that the precuneus is implicated in retrieving and integrating cues related to previous experiences and current addictions, and transmitting the information to the prefrontal cortex, which is also a part of the reward system (38). These two regions have been reported to be responsible for reward processing of the IGD (19, 30, 39). For example, the orbitofrontal cortex was highly activated in the case of winning money, and the activation of anterior cingulate gyrus was decreased in the case of losing, which indicates that the individuals with IGD are sensitive to reward and experience reduced sensitivity to losing (30). A study that tracks substance addiction has found that increased ReHo values in the orbitofrontal cortex of the relapsing heroin addicts, which may indicate abnormal expectations of reward, and the OFC may play an important role in maintaining addictions (21). Research found increased ReHo in the frontal gyrus and precuneus in IGD individuals, which may suggest enhanced reward function in IGD individuals(40). Further, results showed that there was a significant positive correlation between the precuneus and self-reported craving in the PER-IGD individuals compared to RE-IGD individuals, which indicates that the precuneus may regulate the degree of craving for games and affect withdrawal related to addiction. Hence, in this study, we speculated that the increased ReHo values of the OFC and the precuneus may indicate the reward processing ability of PER-IGD individuals was impaired, and which may affect the natural recovery from IGD.

### Increased ReHo values in inhibitory control -related regions in the PER-IGD individuals

This study finds the ReHo value of the DLPFC was higher in the PER-IGD individuals compared to RE-IGD individuals. The DLPFC is a part of the executive control network (41, 42), which plays an important role in executive control and cognitive flexibility(43, 44). It has been implicated in inhibitory control and may be linked importantly to the development and maintenance of addictions (45–48). In a cue-reactivity task, the DLPFC of the IGD individuals was significantly activated when they saw game-related cues, which may suggest that the IGD individuals showed attention bias to game-related cues and reduced the self-inhibition ability(19). Our previous cue-craving task revealed that the DLPFC may affect the recovery from IGD (8). A study also found significant activation of the DLPFC in those addicted to cigarettes when they faced cigarette-related cues (49). A study about gambling disorder linked transcranial direct current stimulation of the DLPFC to improvement in gambling behaviors, suggesting that the DLPFC may be a key component in behavioral addictions (50). Hence, we speculated that the increased ReHo in the DLPFC may represent the impairment of inhibition and control ability of the PER-IGD individuals, and the DLPFC may be linked importantly to the development and maintenance of addictions.

### Decreased ReHo values in audio visual -related regions in the PER-IGD individuals

At the same time, another finding from our study is that decreased ReHo values in the middle temporal gyrus and the postcentral gyrus of PER-IGD individuals compared to RE-IGD individuals. First, the middle temporal gyrus is a part of the temporal lobe, which is responsible for auditory information, complex sound (51) and memory processing (44, 52). Second, the postcentral gyrus is the main somatosensory area, which is responsible for sensory reception and processing of various sensory information (51). Researchers found that these two regions affect the sensitivity to games and augment addiction to games in IGD individuals (45, 53). Studies showed that decreased ReHo in the middle temporal gyrus and the postcentral gyrus internet game addicts, indicating decreased visual and auditory sensitivity (54). Game processing requires intense attention to small changes in the game’s graphics and prolonged exposure to game sounds. The brain areas related to visual and auditory of IGD individuals were stimulated by games for a long time, which increases their tolerance to games and more addicted to games. In this study, we speculated that the decreased ReHo in these two regions may represent the PER-IGD individuals lose the sensitivity to internet games, which may let individuals more addicted to games.

### The brain regions related to reward processing and inhibitory control were changed in functional and structural MRI in the PER-IGD individuals

To further confirm resting-state results, we collected functional MRI and structural data. First, we found that the activity of DLPFC, anterior cingulate gyrus, and insula was significantly higher in the PER-IGD individuals. Second, the structural data results show that the gray matter volume or thickness in the OFC, precuneus, and superior frontal gyrus were decreased in PER-IGD individuals compared to RE-IGD individuals. Research has found that these brain regions may be involved in reward processing and inhibitory control, and were associated with development and maintenance of addictions (28, 34). The PER-IGD individuals may have a high desire for gaming and their inhibitory control is reduced, which affects the withdrawal related to addiction. The structural and functional MRI results further support the results of our resting-state study that PER-IGD individuals showed impairment in some brain regions related to reward processing and inhibitory control, which may affect the natural recovery from IGD.

## Conclusion

This study explored the brain regions related to natural recovery from IGD in a resting-state. The results revealed impairment of brain regions associated with reward processing and inhibitory control (including the OFC, the precuneus, and the DLPFC) of the individuals with PER-IGD, which may be linked importantly to the development and maintenance of addictions. Further, regions related to the visual and auditory system were impaired in the PER-IGD individuals, which may be involved in the addiction to game severity. The structural and functional MRI results further supported our resting-state results. Hence, we suggest that these brain regions may affect the natural recovery from IGD and play an important role in treatments for IGD.

### Limitations

First, the number of participants is limited because of the expensive cost with fMRI studies and the loss of participant over time. We should expand the sample size in future studies. Second, considering the measure of life events is not validated and relatively blunt, therefore warranting a more detailed assessment of life events using validated instruments. Third, male and female subjects were included in this study, but there were relatively few female subjects. Future studies can focus on the differences between male and female subjects in the withdrawal of IGD. Fourth, the explanations are based on reasoning, resulting in some ambiguity in the interpretation.

## Data availability statement

The raw data supporting the conclusions of this article will be made available by the authors, without undue reservation.

## Ethics statement

This study complied with the Declaration of Helsinki and was approved by the Ethics Committee of Wenzhou Medical University. The patients/participants provided their written informed consent to participate in this study.

## Author contributions

XL and YZ designed the research and wrote the first draft of the manuscript. QL and AY analyzed the data and prepared the figures and tables. ZG and PL contributed in collection and preparation of the data. MN, GD, YL, LC, and DX contributed in editing, interpretation, and revision processes. All authors contributed to the article and approved the submitted version.

## Footnotes

1
http://www.restfmri.net/forum/

2
http://www.fil.ion.ucl.ac.uk/spm

3
http://surfer.nmr.mgh.harvard.edu/
