# Supplementary material for: The involvement of spontaneous brain activity in natural recovery from internet gaming disorder: A resting-state fMRI study
Source: Front Psychiatry. 2023 Feb 21;14:1093784. doi: 10.3389/fpsyt.2023.1093784 (PMC9990821; doi:10.3389/fpsyt.2023.1093784)
Supplement: Supplementary file 1 [file Data_Sheet_1.doc]

**Supplementary materials**

**Life events and coding**

In the tracking survey (one year later), additional life events during this year were investigated including family support, free time, changes in romantic relationships, occupations, study habits, aspects of gaming, body health problems, and opinions regarding technologies.

Presence or absence of life events during this period of time (a year) from all subjects was co-varied during second level analyses. The coding was as follows: happened: 1; not happened: 0.
